# Sentiment polarity in nursing notes predicts perioperative complications and shorter hospital stay in hip arthroplasty: Subgroup-specific associations and mediation by complications

**DOI:** 10.1371/journal.pone.0335637

**Published:** 2025-10-30

**Authors:** Li-li Weng, Yan Ni, Zhou-ying Jian, Yi-bin Zhu, Xiao-ling Huang

**Affiliations:** Nursing Department, Sir Run Run Shaw Hospital, Zhejiang University School of Medicine, Hangzhou, China; Kampala International University - Western Campus, UGANDA

## Abstract

**Background:**

Recent advancements in natural language processing (NLP) technology have enabled the extraction of sentiment information from nursing notes. This study aims to investigate the association between sentiment scores and perioperative complications (POC), as well as the length of stay (LOS) in hospital stay, among patients undergoing hip arthroplasty (HA).

**Methods:**

A total of 382 patients undergoing HA were enrolled for this retrospective study, with a POC rate of 22.17% and a median LOS in hospital of 3.56 days. First, the variance inflation factor (VIF) was calculated to perform multicollinearity diagnostics. Subsequently, a restricted cubic spline (RCS) curve was fitted to evaluate the linear relationship between sentiment scores and the risk of POC. Next, multiple logistic regression models were constructed for association analysis. Furthermore, subgroup analyses were conducted to identify susceptible populations, while mediation analysis was employed to explore the mediating role of sentiment scores. Finally, we performed a series of analyses focusing on the secondary outcome of LOS in hospital.

**Results:**

After adjusting for covariates (Model 1 adjusted for age and hypertension; Model 2 adjusted for some laboratory indicators), elevated sentiment polarity scores reduced the risk of POC (Model 1: OR=0.227, 95%CI: 0.062–0.840; Model 2: OR=0.219, 95%CI: 0.058–0.8214). However, this association was no longer significant after adjusting for the reason of surgery (OR=0.587, 95%CI: 0.152–2.319). After adjusting for the operation reason, the association between sentiment polarity and POC was observed in three subgroups: left HA patients, those without dyslipidemia, and those not taking aspirin (all *P* < 0.05). We did not observe the mediating effect of sentiment polarity (all *P* for IE > 0.05). This study also found that higher polarity scores were significantly associated with shorter hospital LOS (β = −2.119, 95%CI: −3.113, −1.124), with POC serving as a mediator in the relationship between sentiment polarity and hospital LOS in both the overall population and the non-dyslipidemia subgroup (*P* for IE < 0.05).

**Conclusion:**

Our findings indicated that sentiment polarity in nursing notes can serve as a valuable predictor of perioperative outcomes in patients undergoing HA.

## 1. Introduction

Hip arthroplasty (HA) is widely recognized as a reliable and appropriate surgical procedure for restoring patient function [1]. The number of HA has been steadily increasing annually, primarily due to the expanding elderly population, driven by increased life expectancy, which necessitates more such procedures [2]. Approximately 450,000 HA procedures are performed each year in the United States, with the case volume continuing to grow [3]. It is estimated that the incidence of primary HA will increase by 71% by 2030 [4]. Although a successful and well-established procedure, HA is coupled with various complications due to the use of prostheses or other fixation devices [5]. The most common major complications following the procedure include mortality, infection, dislocation, revision surgery, and pulmonary embolism [6]. A study conducted in the United States reported a relatively high incidence of perioperative complications (POC) following HA surgery, which were associated with multiple factors [7]. To facilitate more effective management, it is crucial for clinicians to accurately and promptly predict the prognosis of HA patients.

Previous studies have demonstrated that clinicians are capable of predicting mortality in the intensive care unit (ICU), indicating that their notes hold significant value for assessing patients’ health conditions [8–10]. The sentiments in clinical notes can reflect clinicians’ attitudes or impressions regarding patients, which can be quantified and analyzed through sentiment analysis. Sentiment analysis refers to a method used to quantify or classify the subjective characteristics of written text [11,12], encompassing two key indicators: sentiment polarity and sentiment subjectivity. Sentiment polarity refers to the sentiment tendency conveyed in the text, which can be classified as positive or negative, while sentiment subjectivity measures whether the text reflects personal feelings, views, or beliefs [13]. Higher sentiment polarity scores and subjectivity scores indicate greater positivity and subjectivity, respectively. Several studies have revealed that sentiments measured in clinical notes are associated with readmission rates and mortality [14,15]. Especially the sentiment polarity has been found to be a highly significant predictor of 30-day mortality in ICU patients [16]. However, little is known about whether sentiment scores, including sentiment polarity scores and sentiment subjectivity scores, measured in nursing notes are associated with POC or the length of stay (LOS) in hospital following HA.

Therefore, this study aims to evaluate the potential association of sentiment scores with POC and hospital LOS in HA patients. We hypothesize that a higher sentiment score is associated with both a reduced risk of POC and a shorter LOS in hospital following HA.

## 2. Methods

### 2.1. Data source and study participants

Our data source was derived from the Medical Information Mart for Intensive Care (MIMIC-IV) database (version 2.2). MIMIC-IV is an open-access medical database collaboratively created by the Massachusetts Institute of Technology (MIT) and Beth Israel Deaconess Medical Center. This database comprises anonymized clinical information for over 50,000 ICU patients between 2008 and 2019. It encompasses both structured and unstructured data, including vital signs, lab results, medication histories, imaging reports, nursing notes, and diagnostic codes (e.g., ICD codes), spanning a wide range of ages, conditions, and treatment contexts. To protect patient privacy, all identifiable information was replaced with randomized codes. Consequently, neither patient consent nor ethical approval was necessary for this study. The research team has been granted permission to utilize the database and extract data.

**Fig 1:**
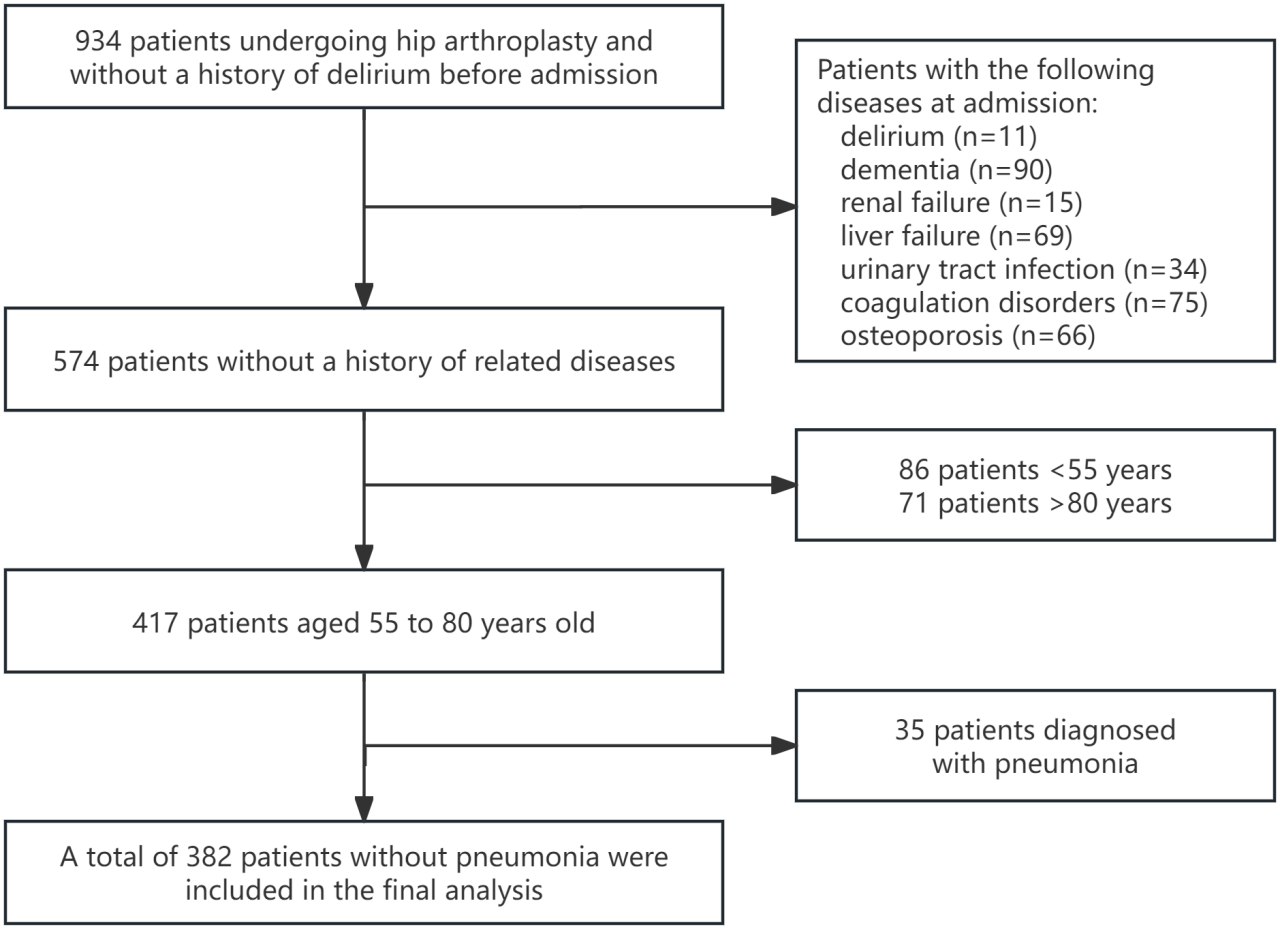
Flowchart of participants selection.

We identified 934 cases of hip joint replacement from the MIMIC-IV database based on ICD-10 codes: 0SRS0JA, 0SRB0J9, 0SRB0JZ, 0SRS0JZ, 0SR904Z, 0SRR03Z, 0SR902Z, 0SR90JZ, 0SR904A, 0SR90JA, 0SRB029, 0SRS0J9, 0SRR0J9, 0SRR03A, 0SRS039, 0SRR0JZ, 0SRR0JA, 0SRS01A, 0SRR01A, 0SR903A, 0SRB0JA, 0SRA0JZ, 0SR901Z, 0SRB01A, 0SR902A, 0SRS03A, 0SRB02A, 0SRB039, 0SR9019, 0SRR039, 0SRB04A, 0SR90J9. After excluding patients diagnosed with delirium (n = 11), dementia (n = 90), renal failure (n = 15), liver failure (n = 69), urinary tract infection (n = 34), coagulation disorders (n = 75), osteoporosis (n = 66), and pneumonia (n = 35) at admission, as well as those under the age of 55 (n = 86), and over the age of 80 (n = 71), a total of 382 patients were identified for further analysis (as shown in **Fig 1**).

### 2.2. Clinical outcomes

In this study, POC in patients undergoing hip replacement surgery was the primary outcome, and the length of stay (LOS) in hospital was the second outcome.

### 2.3. Sentiment analysis

The sentiment analysis of the nursing notes was conducted using TextBlob, a Python library for processing textual data. It is an English text processing toolkit based on the Natural Language Toolkit (NLTK) and Pattern, used for in-depth research on common Natural Language Processing (NLP) tasks, such as part-of-speech tagging, noun phrase extraction, sentiment analysis, classification, translation, etc (https://textblob.readthedocs.io/en/dev/). NLTK was employed to divide the nursing notes into several words, and Pattern, which includes dictionaries of various English adverbs and adjectives, can map to three dimensions of sentiment scores: polarity, subjectivity, and intensity [10,16]. It can identify these words in the nursing notes and calculate the corresponding scores. In this research, the sentiment polarity scores and subjectivity scores derived from each nursing note were utilized as independent variables.. Sentiment polarity score is quantified as a continuous value ranging from −1–1, whereas subjectivity score is expressed as a value between 0 and 1 [8]. Higher scores indicated a more positive and subjective sentiment, respectively [16]. Examples were provided in S1 Table.

### 2.4. Data collection

This study also extracted data from five major domains: [1] Demographic data, including age, gender, race, marital status, insurance, smoking, drinking, and body mass index (BMI). [2] Surgical-related variables, including transfusion, reason of operation, surgical site, and bone cemented. [3] Comorbidities, such as dyslipidemia, diabetes, hypertension, congestive heart failure (CHF), and Charlson Comorbidity Index (CCI). [4] Medication use, including tramadol, warfarin, and aspirin. [5] Laboratory indicators, including anion gap (AG), bicarbonate, blood urea nitrogen (BUN), calcium, chloride, creatinine, hematocrit, hemoglobin, potassium, red blood cell (RBC), red blood cell distribution width (RDW), sodium, white blood cell (WBC), phosphate, glucose, and platelets.

### 2.5. Statistical analysis

Continuous variables were reported as mean ± standard deviation (SD) for those that followed a normal distribution, or as median (interquartile range, IQR) for those that did not. Categorical variables were summarized as counts (percentages). In the analysis of continuous data, t-tests or ANOVA were employed for normally distributed datasets, whereas the Kruskal-Wallis test or Mann-Whitney U test was utilized for non-normally distributed datasets. Categorical data were evaluated using chi-square tests or Fisher’s exact tests, depending on the suitability of the data. All statistical analyses were performed using SPSS version 26.0 and R version 4.2.1, and *P* < 0.05 (two-sided) was considered statistically significant.

First, we compared the different variables between the non-POC group and the POC group. The multicollinearity among variables with differences between groups was examined on the basis of the variance inflation factor (VIF). Second, a restricted cubic spline (RCS) curve was fitted to investigate whether the relationship between sentiment polarity and the risk of POC was linear. Logistic regression predicts binary outcomes by modeling probabilities through a logistic function with predictor variables [17]. Therefore, the relationship between sentiment polarity and POC risk was examined using four distinct logistic regression models based on data imputed via interpolation. The crude model did not adjust for any variables. Model 1 adjusted for age and hypertension; model 2 adjusted for AG, bicarbonate, and hemoglobin; model 3 adjusted for reason of operation. Third, further analysis was performed to examine the baseline characteristics that varied among groups with different surgical reasons. Stratified analysis was performed on the indicators exhibiting intergroup differences to investigate the association between sentiment polarity and POC after adjusting for the reason of operation. Four, an analysis was performed in subgroups stratified by patient age (≤60 or >60) at admission, hypertension status (with or without), and reason of operation (tibial fracture, osteoarthritis, or other) to investigate whether significant differences existed in the polarity scores. Furthermore, we conducted a mediation analysis to examine whether the three variables influenced the polarity score, thereby leading to POC. Finally, we employed the generalized linear regression model to assess the association between sentiment polarity and the secondary outcome of LOS in hospital. Additionally, we conducted a mediation analysis to investigate whether the polarity score influenced LOS in hospital by affecting the occurrence of complications. E-value was proposed to assess potential unmeasured or residual confounding in observational studies and its calculation formula is as follows: OR (odds ratio) = OR + sqrt {OR* (OR-1)} [18]. Hence, we evaluated the robustness of results by calculating the E-value.

## 3. Results

### 3.1. Descriptive results

A total of 382 patients who underwent HA were included, of whom 63 had POC. The median age was 66 years, and the proportion of females was 61.00% (**Table 1**). Patients with POC were older, had a more evenly distributed proportion of surgical reasons, a higher proportion of hypertension, a longer hospital stay, and a lower sentiment polarity score (all *P* < 0.05). Higher levels of AG, bicarbonate, hematocrit, and hemoglobin were observed in POC patients (all *P* < 0.05). There was no significant difference in gender, race, marital status, insurance, smoking, drinking, BMI, transfusion, surgical site, bone cemented, dyslipidemia, diabetes, CHF, CCI, tramadol, warfarin, aspirin, BUN, calcium, chloride, creatinine, potassium, RBC, RDW, sodium, WBC, phosphate, glucose, platelets, and sentiment subjectivity (all *P* > 0.05).

**Table 1:** Comparison of baseline characteristics according to incidence of POC.

Variable	Overall (n = 382)	Non-POC (n = 319)	POC (n = 63)	*P*-value
Age, years	66.000[61.000,73.000]	66.000[61.000,72.000]	69.000[63.000,76.000]	0.025
BMI, kg/m^2^	27.700[24.400,32.400]	27.800[24.600,32.500]	27.100[24.200,30.800]	0.251
Gender, n (%)				0.687
Male	149 (39.005)	123 (38.558)	26 (41.270)	
Female	233 (60.995)	196 (61.442)	37 (58.730)	
Race, n (%)				0.919
White	316 (83.158)	265 (83.072)	51 (83.607)	
Others	64 (16.842)	54 (16.928)	10 (16.393)	
Marital status, n (%)				0.781
Married	190 (50.000)	160 (50.314)	30 (48.387)	
Other	190 (50.000)	158 (49.686)	32 (51.613)	
Insurance, n (%)				0.554
Medicare	207 (54.188)	175 (54.859)	32 (50.794)	
Other	175 (45.812)	144 (45.141)	31 (49.206)	
Smoking, n (%)				0.263
No	376 (98.429)	315 (98.746)	61 (96.825)	
Yes	6 (1.571)	4 (1.254)	2 (3.175)	
Drinking, n (%)				0.168
No	373 (97.644)	313 (98.119)	60 (95.238)	
Yes	9 (2.356)	6 (1.881)	3 (4.762)	
Transfusion, n (%)				0.056
No	354 (92.670)	292 (91.536)	62 (98.413)	
Yes	28 (7.330)	27 (8.464)	1 (1.587)	
Reason of operation, n (%)				<0.001
Tibial fracture	51 (13.351)	27 (8.464)	24 (38.095)	
Osteoarthritis	228 (59.686)	207 (64.890)	21 (33.333)	
Other	103 (26.963)	85 (26.646)	18 (28.571)	
Surgical site, n (%)				0.272
Left hip	176 (46.073)	143 (44.828)	33 (52.381)	
Right hip	206 (53.927)	176 (55.172)	30 (47.619)	
Bone cemented, n (%)				0.333
No	193 (82.833)	166 (83.838)	27 (77.143)	
Yes	40 (17.167)	32 (16.162)	8 (22.857)	
Dyslipidemia, n (%)				0.479
Yes	33 (8.639)	29 (9.091)	4 (6.349)	
Diabetes, n (%)				0.425
Yes	38 (9.948)	30 (9.404)	8 (12.698)	
Hypertension, n (%)				0.034
Yes	104 (27.225)	80 (25.078)	24 (38.095)	
CHF, n (%)				0.849
Yes	34 (8.901)	28 (8.777)	6 (9.524)	
CCI	4.000[3.000,5.000]	4.000[3.000,5.000]	4.000[4.000,5.000]	0.145
Tramadol, n (%)				0.103
Yes	63 (16.492)	57 (17.868)	6 (9.524)	
Warfarin, n (%)				0.697
Yes	26 (6.806)	21 (6.583)	5 (7.937)	
Aspirin, n (%)				0.18
Yes	181 (47.382)	156 (48.903)	25 (39.683)	
Admission index				
AG, mEq/L	13.000[12.000,15.000]	13.000[12.000,15.000]	14.000[12.000,16.000]	0.017
Bicarbonate, mEq/L	25.000[24.000,27.000]	26.000[24.000,27.000]	24.000[22.000,26.000]	0.008
BUN, mg/dL	15.000[12.000,21.000]	15.000[12.000,20.000]	18.000[12.000,24.000]	0.266
Calcium, mg/dL	8.500[8.200,8.900]	8.500[8.200,8.900]	8.600[8.300,8.900]	0.726
Chloride, mEq/L	101.000[99.000,104.000]	101.000[99.000,104.000]	102.000[99.000,104.000]	0.792
Creatinine, mg/dL	0.800[0.700,1.000]	0.800[0.700,1.000]	0.900[0.700,1.000]	0.711
Hematocrit, %	32.876 ± 4.863	32.620 ± 4.779	34.190 ± 5.075	0.020
Hemoglobin, g/dL	10.730 ± 1.702	10.634 ± 1.676	11.223 ± 1.747	0.013
Potassium, mEq/L	4.100[3.800,4.400]	4.100[3.800,4.500]	4.100[3.800,4.400]	0.643
RBC, m/μL	3.576 ± 0.542	3.550 ± 0.539	3.698 ± 0.540	0.059
RDW, %	13.400[12.700,14.300]	13.300[12.700,14.200]	13.500[12.700,14.500]	0.258
Sodium, mEq/L	138.000[136.000,140.000]	138.000[136.000,140.000]	139.000[136.000,140.000]	0.704
WBC, K/μL	10.000[8.200,12.400]	10.000[8.200,12.400]	9.500[8.000,12.300]	0.675
Phosphate, mg/dL	3.451 ± 0.693	3.451 ± 0.674	3.447 ± 0.767	0.968
Glucose, mg/dL	124.000[109.000,139.000]	125.000[110.000,140.000]	122.000[104.000,131.000]	0.134
Platelets, K/μL	188.000[149.000,223.000]	189.000[150.000,223.000]	180.000[146.000,213.000]	0.217
LOS in hospital, day	3.556[2.635,4.538]	3.520[2.599,3.990]	4.303[3.559,5.677]	<0.001
Sentiment polarity	0.060[0.050,0.072]	0.061[0.051,0.072]	0.056[0.038,0.068]	0.026
Sentiment subjectivity	0.312[0.291,0.332]	0.312[0.293,0.332]	0.310[0.279,0.332]	0.103
Scaled sentiment polarity	0.605[0.501,0.717]	0.614[0.507,0.720]	0.562[0.381,0.685]	0.026

Median [IQR] or mean ± SD for continuous variables and counts (percentage) for categorical variables. Abbreviations: POC, perioperative complication; IQR, interquartile range; SD, standard deviation; BMI, body mass index; CHF, congestive heart failure; AG, anion gap; BUN, blood urea nitrogen; RBC, red blood cell; RDW, red blood cell distribution width; WBC, white blood cell; LOS, length of stay; CCI, Charlson Comorbidity Index.

The missingness of the variables in this study was summarized in S2 Table. The proportion of missing data in this study ranges from 0 to 39%, and no data was excluded due to a high proportion of missing values.

### 3.2. Associations between sentiment polarity and POC in patients undergoing HA

**Fig 2:**
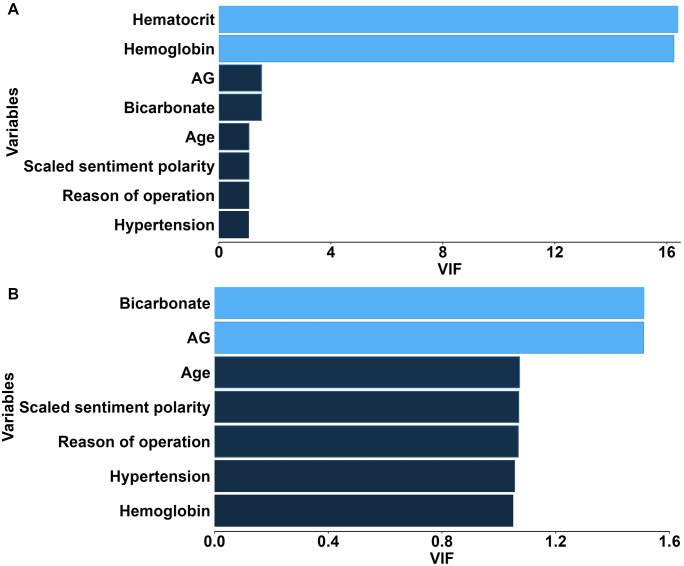
Multicollinearity analysis through calculating VIF. **(A)** The VIF of the variables with significant differences among the groups; **(B)** VIF after hematocrit exclusion. Abbreviations: VIF, variance inflation factor; AG, anion gap.

**Fig 3:**
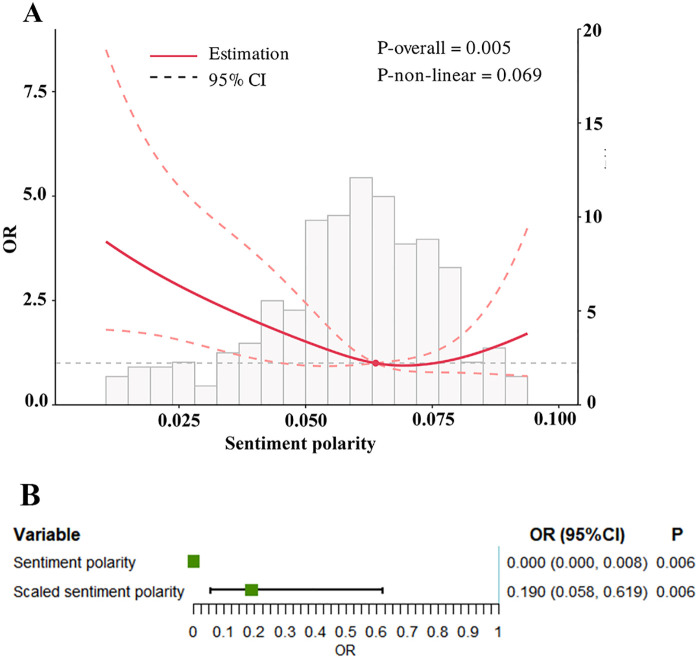
Association between sentiment polarity and POC. **(A)** Restricted cubic spline (RCS) curve analysis; **(B)** Univariate logistic regression analysis. Abbreviations: POC, perioperative complication; OR, odds ratio; CI, confidence interval.

Since no significant difference in sentiment subjectivity was observed between the two groups, the subsequent analysis concentrated on sentiment polarity. After excluding the variable ‘hematocrit’ with the highest VIF value (VIF = 16.39, **Fig 2A**), no multicollinearity was found among the remaining covariates (all VIF < 2), so they were included in the subsequent analysis (**Fig 2B**). To investigate the dose-response relationship between sentiment polarity and POC, we fitted the RCS curve. The curve initially exhibited a declining trend, followed by a subsequent rise (**Fig 3A**). There was a linear relationship between sentiment polarity and POC (*P* for nonlinear >0.05). Thus, a logistic regression model was established to investigate the association between sentiment polarity and the risk of POC. We found that the *P* value indicated a significant effect (*P* = 0.006), but the odds ratio (OR) was abnormal (OR: 0, 95%confidence interval [CI]: 0–0.008) (**Fig 3B**). The OR value reflects the change in risk associated with each one-unit increase or decrease. In this study, the independent variable was the sentiment polarity score, which ranged from −1 to 1, representing a total variation of 2 units. Consequently, interpreting the OR value becomes challenging, as a one-unit change corresponds to half of the entire range of the polarity score, potentially introducing bias and resulting in an OR that is very small and difficult to interpret. Therefore, based on prior literature [16], this study multiplied the sentiment polarity variable by 10 (scaled sentiment polarity) for logistic regression analysis under the assumption of a linear relationship. After this adjustment, both the OR value and its confidence interval became more reasonable (OR: 0.190, 95%CI: 0.058–0.619) (**Fig 3B**). Given that some laboratory indicators contain a substantial number of missing values, interpolation was employed to impute these missing values prior to subsequent analyses.

**Fig 4:**
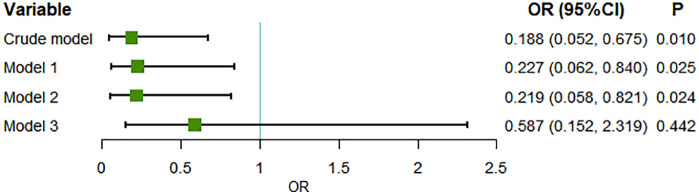
Association between sentiment polarity and POC after imputation in different logistic models. Note: the crude model did not adjust for any variables; model 1 adjusted for age and hypertension; model 2 adjusted for AG, bicarbonate, and hemoglobin; model 3 adjusted for reason of operation. Abbreviations: POC, perioperative complication; OR, odds ratio; CI, confidence interval; AG, anion gap.

Next, four different logistic regression models were established to investigate the association between scaled sentiment polarity and POC (**Fig 4**). The results indicated that a higher scaled sentiment polarity score was associated with a reduced risk of POC in patients undergoing HA. In the crude model, OR was 0.188 (95%CI: 0.052–0.675). After adjusting for age and hypertension (Model 1) and laboratory indicators (Model 2), the association remained significant with the ORs of 0.227 (95% CI: 0.062–0.840) and 0.219 (95%CI: 0.058–0.8214), respectively. However, the association did not exist in Model 3 (adjusted for reason of operation), with an OR of 0.587 (95%CI: 0.152–2.319). To assess the robustness of our findings, we additionally performed an analysis using non-imputed data, which yielded largely consistent results (S3 Table). Moreover, we assessed the robustness of the results through calculating E-values, and the results confirmed the robustness of our main findings (E-values for crude model and model 1–3 were 10.11, 8.28, 8.60 and 2.80, respectively).

Following adjustment for the reason of surgery, the association was no longer observed. We propose three potential explanations for this finding. First, the reason of operation may serve as a confounding factor influencing the association between polarity and POC. However, no significant interaction was detected in the interaction analysis (*P* > 0.05), nor was any association observed after stratification by surgical reason (*P* > 0.05). Second, collinearity among variables might exist; however, prior analyses have confirmed the absence of collinearity. Third, the limited sample size could be a contributing factor. Nevertheless, it is not feasible to increase the sample size or validate the finding using external data.

**Fig 5:**
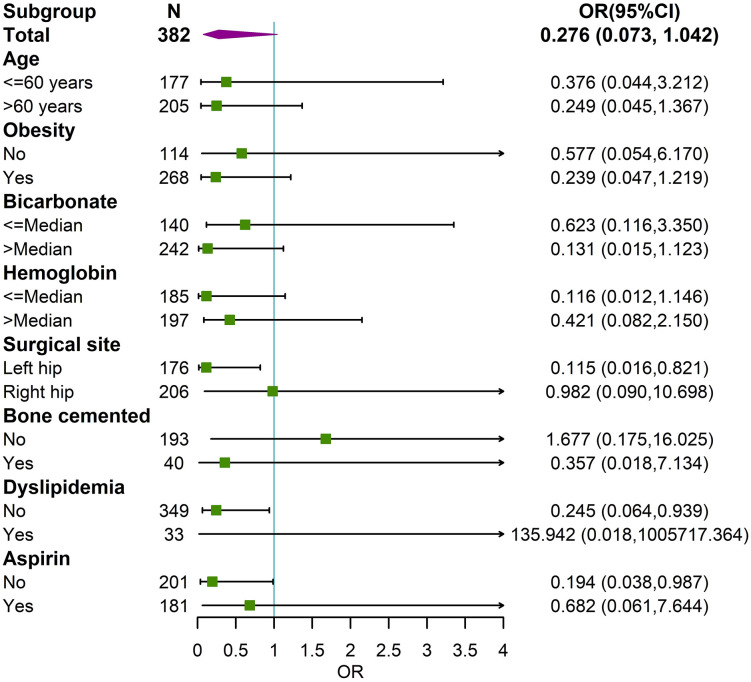
Subgroup analyses between sentiment polarity and POC after adjusting for the reason of operation. Abbreviations: POC, perioperative complication; OR, odds ratio; CI, confidence interval.

We further speculate whether there are some undetected differences among subgroups with different surgical reasons, which may obscure the association between polarity and POC. The results indicated significant inter-group differences in the following 8 variables (**Table 2**): age, BMI, surgical site, bone cemented, dyslipidemia, aspirin, bicarbonate, and hemoglobin (**P* *< 0.05). Subgroup analysis stratified by these 8 variables revealed that (**Fig 5**), after adjusting for reason of operation, the association between polarity and POC became statistically significant in three specific subgroups: patients undergoing left HA, those without dyslipidemia, and those not taking aspirin (*P* < 0.05). In the total population, sentiment polarity cannot be considered an independent predictor of POC due to confounding bias introduced by the reason of operation. However, in the subgroup populations—including patients undergoing left HA, those without dyslipidemia, or those not taking aspirin—sentiment polarity independently predicted the risk of POC.

**Table 2:** Comparison of baseline characteristics according to the reason of operation.

Variable	Overall (n = 382)	Tibial fracture (n = 51)	Osteoarthritis (n = 228)	Other (n = 103)	*P*-value
Age, years	66.000[61.000,73.000]	73.000[65.000,78.000]	65.000[60.000,72.000]	67.000[61.000,71.000]	<0.001
BMI, kg/m^2^	27.700[24.400,32.400]	25.100[22.900,28.700]	28.700[24.700,33.200]	27.800[24.900,32.300]	0.004
Surgical site, n (%)					0.030
Left hip	176 (46.073)	32 (62.745)	102 (44.737)	42 (40.777)	
Right hip	206 (53.927)	19 (37.255)	126 (55.263)	61 (59.223)	
Bone cemented, n (%)					<0.001
No	193 (82.833)	24 (68.571)	119 (90.840)	50 (74.627)	
Yes	40 (17.167)	11 (31.429)	12 (9.160)	17 (25.373)	
Dyslipidemia, n (%)					
Yes	33 (8.639)	1 (1.961)	28 (12.281)	4 (3.883)	0.008
Aspirin, n (%)					
Yes	181 (47.382)	10 (19.608)	115 (50.439)	56 (54.369)	<0.001
Admission index					
Bicarbonate, mEq/L	25.000[24.000,27.000]	24.000[22.000,27.000]	26.000[24.000,27.000]	25.000[23.500,27.000]	0.03
Hemoglobin, g/dL	10.738 ± 1.702	11.441 ± 1.655	10.608 ± 1.601	10.678 ± 1.853	0.006
Scaled sentiment polarity	0.605[0.501,0.717]	0.443[0.224,0.583]	0.618[0.529,0.710]	0.636[0.518,0.757]	<0.001

Median [IQR] or mean ± SD for continuous variables and counts (percentage) for categorical variables. Abbreviations: IQR, interquartile range; SD, standard deviation; BMI, body mass index.

**Fig 6:**
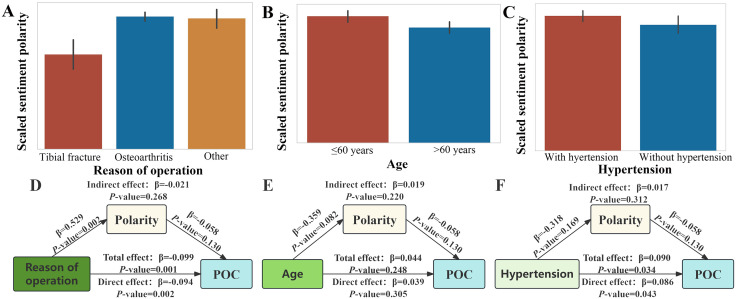
Differences in sentiment polarity across different baseline characteristics and mediation analyses of sentiment polarity. **(A)** Reason of operation (*P* < 0.001); **(B)** Age (*P* = 0.032); **(C)** Hypertension (*P* = 0.040); **(D)** Reason of operation; **(E)** Age; **(F)** Hypertension. Abbreviations: POC, perioperative complication.

Furthermore, we also raised the question of whether baseline differences may affect POC by influencing polarity. The baseline data regarding age, hypertension, and reason of operation showed differences between POC and non-POC groups. As shown in Fig 6A-C, we observed significant differences in sentiment polarity scores across the subgroups stratified by these 3 variables (all *P* < 0.05). Subsequently, we conducted a mediation analysis to investigate whether these variables influenced the outcome indirectly by affecting the sentiment polarity scores. **Fig 6D** and **Fig 6F** showed that surgical reason and hypertension were both associated with POC (*P* for total effect [TE] <0.05), but sentiment polarity didn’t act as a mediator (indirect effect [IE]: β = −0.021, *P* = 0.268; IE: β = 0.017, *P* = 0.312). We further observed that age was not significantly associated with POC (*P* for TE > 0.05), and polarity did not serve as a mediator in the pathway (IE: β = 0.019, *P* = 0.220) (**Fig 6D**).

### 3.3. Association between sentiment polarity and LOS in hospital: the mediating role of POC

**Fig 7:**
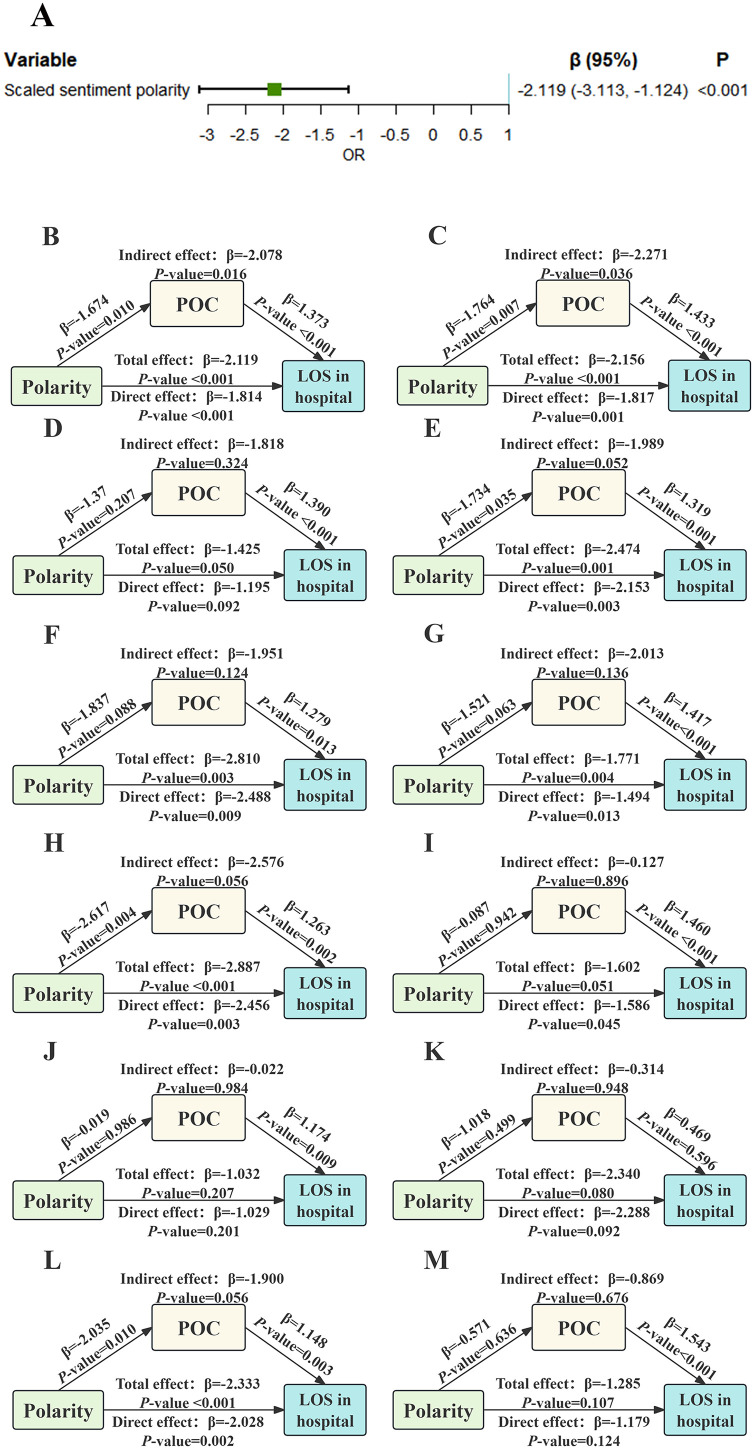
Summary of secondary outcome analysis results. **(A)** Association between sentiment polarity and LOS in hospital; **(B)** Mediation analysis for the overall population; **(C)** Mediation analysis for patients without dyslipidemia; **(D-E)** Mediation analysis for patients under 60 years old or over 60 years old; **(F-G)** Mediation analysis for non-obese versus obese patients; (H-I) Mediation analysis for patients undergoing left HA versus right HA; **(J-K)** Mediation analysis for non-cemented group versus cemented group; **(L-M)** Mediation analysis for patients not taking aspirin versus taking aspirin. Abbreviations: POC, perioperative complication; LOS, length of stay; HA, hip arthroplasty.

Finally, we examined the association between scaled sentiment polarity and the secondary outcome of LOS in hospital (**Fig 7A**). Our findings indicated that higher polarity scores were significantly associated with shorter hospital LOS (β = −2.119, 95%CI: −3.113, −1.124). Subsequently, we conducted mediation analyses to investigate whether sentiment polarity influenced LOS in hospital indirectly through its effect on complications. This analysis was performed in both the overall population and the sub-population. In the overall population (**Fig 7B**), POC served as a partial mediator (IE: β = −2.078, *P* = 0.016; DE: β = −1.814, **P* *< 0.001) in the sentiment polarity-hospital LOS pathway. In the subgroup of individuals without dyslipidemia (**Fig 7C**), POC served as a partial mediator (IE: β = −2.271, *P* = 0.036; DE: β = −1.817, *P = *0.001). In the remaining subgroups (Fig 7D-M), no significant mediating effect of POC was detected (all *P* for IE > 0.05).

## 4. Discussion

The results show that sentiment polarity is significantly associated with POC in HA patients even after adjusting for various confounders. However, after adjusting for the reason of operation, the significant association disappeared. Additionally, subgroup analysis revealed that, after adjusting for the operation reason, the association between sentiment polarity and POC was observed in three subgroups: left HA patients, those without dyslipidemia, and those not taking aspirin. We did not observe the mediating effect of sentiment polarity. This study also found that higher polarity scores were significantly associated with shorter hospital LOS, with POC serving as a mediator in the relationship between sentiment polarity and hospital LOS in both the overall population and the non-dyslipidemia subgroup.

The sentiment scores in nursing notes are like a mirror, which can not only reflect the potential harm caused to patients by the attitudes of healthcare providers, but also reveal the complex predicaments of patients themselves that are closely linked to poor prognosis. Firstly, the negative attitude of medical staff can reduce patients’ understanding and trust in treatment, thereby leading to a decline in patient compliance [19]. For instance, a retrospective correlational study conducted in Italy indicates that a physician’s positive attitude and empathetic communication are significantly associated with improved clinical outcomes in patients with diabetes mellitus [20]. Secondly, patients with severe conditions, difficult-to-control symptoms, or poor treatment responses may inherently cause frustration, helplessness, or anxiety among medical staff, resulting in more negative emotions being expressed in the notes [21]. Finally, in an environment with limited resources, heavy workloads, and high stress, medical staff are more prone to negative emotions, which may lead to a decrease in patience and attention to detail for all patients [22,23]. This systemic issue can affect the overall quality of care and indirectly contribute to more adverse outcomes for patients.

The findings of this study demonstrated a significant association between sentiment polarity and POC, whereas sentiment subjectivity did not exhibit a statistically significant effect. Sentiment polarity captures the directional valence of expressed emotions in text—whether positive, negative, or neutral—while sentiment subjectivity indicates the extent to which the content reflects personal opinions versus objective facts [24]. Although polarity classification is traditionally considered applicable only to subjective texts, emerging evidence suggests that objective texts may also convey emotional directionality [25]. Furthermore, studies have shown that in medical contexts, positively and negatively framed factual statements (i.e., objective information) occur nearly as frequently as affect-laden opinions and experiential reports (i.e., subjective information) [26]. Thus, the lack of a significant association with sentiment subjectivity is understandable, as subjectivity per se does not determine the valence of sentiment and is therefore less indicative of clinical status severity.

In recent years, an increasing number of studies have focused on analyzing sentiments in clinical notes to evaluate their predictive value for patients’ prognosis outcomes [27,28]. These studies have focused more on the mortality rate of ICU patients [8,10,16,29]. However, this study aims to determine the association between sentiments and the risk of POC and LOS in hospital in patients undergoing HA. The results show that sentiment polarity is negatively associated with both the risk of POC and LOS in hospital. A study based on electronic health data from a US hospital showed that greater positive sentiments are significantly associated with a reduced risk of readmission [14]. A lower readmission rate is indicative of a reduced risk of complications, aligning with the findings of this study. Patients with more unstable or difficult-to-control conditions may evoke stronger emotions in clinicians, leading them to use more emotionally charged terms [14], which may well explain the findings of this study.

However, the significant association between sentiment polarity and POC no longer existed after adjusting for the reasons for operation. Further analysis confirmed that the reason for operation was a key confounding factor. Therefore, in the total population, we cannot consider sentiment polarity as an independent predictor of POC, as its ability to predict POC depends on the reason for operation and has no independent incremental value. Subgroup analysis revealed that among patients undergoing left HA, those without dyslipidemia, and those not taking aspirin, sentiment polarity was significantly associated with the risk of POC after adjusting for the reason of operation. This result indicates that sentiment polarity is an independent predictor of POC risk in these subgroups of HA patients. We have proposed several potential explanations for this phenomenon. Studies have shown that surgeon handedness and whether operating on the dominant or non-dominant side could affect the acetabular cup positioning and outcomes during HA [30,31]. Research in other surgical fields, such as breast augmentation, has also indicated that surgeons’ handedness may influence the incidence of complications following these procedures [32]. Given that the majority of surgeons are right-handed, surgical procedures performed on the left side may not be as “natural” or habitual as on the right side [33,34]. This may necessitate greater vigilance, improved coordination, and more deliberate communication among all members of the surgical team, including nursing personnel [35]. Positive affective states within the medical team may facilitate more efficient procedural execution and quicker response times in such high-demand contexts, thereby contributing to a reduced likelihood of complications. In contrast, negative emotions may heighten the propensity for minor errors in these less familiar circumstances, which, when compounded, could elevate the overall risk of adverse outcomes. This could render sentiment polarity a more robust predictor of POC within this subgroup. Aspirin exhibits antiplatelet properties and potent anti-inflammatory effects [36]. Patients not taking aspirin may indicate better overall health status, which could contribute to higher sentiment polarity scores in the clinical staff and lower POC risks in patients themselves. The situation is similar for those without dyslipidemia.

Clinically, these findings suggest that integrating sentiment analysis of nursing notes into preoperative risk assessments could enhance early identification of high-risk patients. Clinicians should consider incorporating measured sentiment as a routinely monitored variable to serve as an indicator of health outcomes for HA patients, particularly in vulnerable subgroups, to optimize perioperative care and resource allocation.

Future longitudinal studies could facilitate the examination of temporal patterns in nursing notes, thereby enhancing our understanding of sentiment nuances. Additionally, various methods exist for measuring sentiments, and different techniques may produce varying results. Future research could use the machine learning-based models for semantic reasoning to improve sentiment analysis. Moreover, if nurses in the future were to assign their notes a “negative”, “positive”, or “neutral” score, this would effectively create a labeled corpus of nursing notes, providing an invaluable resource for advancing sentiment analysis research in medical records [16,37]. It is also a promising approach to develop a gold-standard sentiment dictionary tailored to the unique features of clinical narratives [38].

Several limitations in this study warrant attention. First, this is a retrospective study, and the causality of the relationship between sentiment polarity and clinical outcomes cannot be confirmed. Second, the data in this study were obtained from the MIMIC-IV database, with the study population primarily consisting of American individuals. This introduces geographical and racial limitations, restricting the generalizability of the findings to other populations. Third, residual confounding due to unmeasured variables cannot be entirely ruled out. For example, the amount of text data in nursing notes is influenced by the time available to nurses. When nurses are overburdened with tasks, the volume of textual information may diminish, which can have an impact on the results. Consequently, there is a need for more precise sentiment measurement and external validation. Finally, there is still no gold standard dictionary for sentiment analysis in the medical field [39]. In the future, efforts should be focused on developing a gold standard sentiment dictionary suitable for clinical narratives (such as less neutral content and inconsistent polarity) and combining it with the most advanced machine learning methods for sentiment analysis.

## 5. Conclusion

Sentiment polarity in nursing notes is associated with POC and length of hospital stay among patients undergoing HA. Notably, among patients undergoing left HA, those without dyslipidemia, or those not taking aspirin, sentiment polarity independently predicted POC risk. In addition to traditionally used structured data, unstructured information in nursing notes can also serve as a valuable indicator of clinical outcomes.

## Supporting information

S1 Table Sample excerpts from ICU nursing notes and their corresponding sentiment polarity scores. (DOCX)

S2 Table Proportion of missing data in this analysis. (DOCX)

S3 Table Association between sentiment polarity and POC before and after imputation in different logistic models. (DOCX)
