# Supplementary material for: Sentiment polarity in nursing notes predicts perioperative complications and shorter hospital stay in hip arthroplasty: Subgroup-specific associations and mediation by complications
Source: PLoS One. 2025 Oct 30;20(10):e0335637. doi: 10.1371/journal.pone.0335637 (PMC12574854; doi:10.1371/journal.pone.0335637)
Supplement: S1 Table — (DOCX) [file pone.0335637.s001.docx]

**Table S1** Sample excerpts from ICU nursing notes and their corresponding sentiment polarity scores.

|  | Part of the nursing notes | Polarity score | Subjectivity score |
| --- | --- | --- | --- |
| Patient 1 | Patient lives with her grandmother and aunt. She is G3P2; she has two children and had a spontaneous abortion in ___. On ___ pm, the patient described an extensive history of domestic physical and emotional abuse. She reported that the father of her children abused her physically from ___ and "stomped on her with a boot," though she said the experience had been so terrible she had forgotten where he had hurt her, and whether she had experienced trauma to her epigastric region or sternum. She also describes an abusive relationship approximately ___ years ago; she believes this boyfriend tried to poison her iced tea, which resulted in her hospitalization and an EGD. | 0.026 | 0.419 |
| Patient 2 | Pt is an ___ Y M year old male with Hx of metastatic lung/neuroendocrine tumor s/p ___ + etoposide x6 cycles  ___ to ___ and then 4 more cycles ___ to ___ with disease progression and hepatic metastasis from PET on ___ presents from his oncologist with fatigue and leukocytosis. His WBC 62, Hgb 10.8, Plt 158 on ___ and repeated labs ___ at ___ showed WBC 168, Hgb 11.9 and Plt 106. He was sent to ___ ED where his WBC was 167, Hgb 9.4 and Plt 83. Cr increased to 2.6. Phos 0.7 and Uric acid 19.5. The  patient feels generally very well although he has been somewhat fatigued. He also claims that he has black spots in his vision but no headache, nausea, vomiting, neck stiffness. He has had mild shortness of breath with exertion but denies any cough, chest pain, orthopnea, PND. | 0.072 | 0.420 |
