# Supplementary material for: Sentiment polarity in nursing notes predicts perioperative complications and shorter hospital stay in hip arthroplasty: Subgroup-specific associations and mediation by complications
Source: PLoS One. 2025 Oct 30;20(10):e0335637. doi: 10.1371/journal.pone.0335637 (PMC12574854; doi:10.1371/journal.pone.0335637)
Supplement: S2 Table — (DOCX) [file pone.0335637.s002.docx]

**Table S2** Proportion of missing data in this analysis

| Variable | Proportion of missing value |
| --- | --- |
| Age, years | 0 |
| BMI | 0 |
| Gender | 0 |
| Race | 0.005 |
| Marital status | 0.005 |
| Insurance | 0 |
| Smoking, | 0 |
| Drinking | 0 |
| Transfusion | 0 |
| Reason of operation | 0 |
| Surgical site | 0 |
| Bone cemented | 0.390 |
| Dyslipidemia | 0 |
| Diabetes | 0 |
| Hypertension | 0 |
| CHF | 0 |
| CCI | 0 |
| Tramadol | 0 |
| Warfarin | 0 |
| Aspirin | 0 |
| AG, mEq/L | 0.134 |
| Bicarbonate, mEq/L | 0.134 |
| BUN, mg/dL | 0.136 |
| Calcium, mg/dL | 0.220 |
| Chloride, mEq/L | 0.134 |
| Creatinine, mg/dL | 0.005 |
| Hematocrit, % | 0.005 |
| Hemoglobin, g/dL | 0.005 |
| Potassium, mEq/L | 0.120 |
| RBC, m/μL | 0.134 |
| RDW, % | 0.134 |
| Sodium, mEq/L | 0.134 |
| WBC, K/μL | 0.136 |
| Phosphate, mg/dL | 0.215 |
| Glucose, mg/dL | 0.131 |
| Platelets, K/μL | 0.136 |
| LOS in hospital, day | 0 |
| Sentiment polarity | 0 |
| Sentiment subjectivity | 0 |
| Scaled sentiment polarity | 0 |
