# Supplementary material for: Sentiment polarity in nursing notes predicts perioperative complications and shorter hospital stay in hip arthroplasty: Subgroup-specific associations and mediation by complications
Source: PLoS One. 2025 Oct 30;20(10):e0335637. doi: 10.1371/journal.pone.0335637 (PMC12574854; doi:10.1371/journal.pone.0335637)
Supplement: S3 Table — (DOCX) [file pone.0335637.s003.docx]

**Table S3** Association between sentiment polarity and POC before and after imputation in different logistic models.

|  | Before imputation | | After imputation | |
| --- | --- | --- | --- | --- |
|  | OR(95%CI) | *P*-value | OR(95%CI) | *P*-value |
| Crude model | 0.188(0.052 - 0.675) | 0.010 | 0.188(0.052 - 0.675) | 0.010 |
| Model 1 | 0.227(0.062 - 0.840) | 0.025 | 0.227(0.062 - 0.840) | 0.025 |
| Model 2 | 0.308(0.077 - 1.228) | 0.094 | 0.219(0.058 - 0.821) | 0.024 |
| Model 3 | 0.587(0.152 - 2.319) | 0.442 | 0.587(0.152 - 2.319) | 0.442 |

Note: the crude model did not adjust for any variables; model 1 adjusted for age and hypertension; model 2 adjusted for AG, bicarbonate, and hemoglobin; model 3 adjusted for reason of operation. Abbreviations: POC, perioperative complication; OR, odds ratio; CI, confidence interval; AG, anion gap.
